# Electrically regulated cell‐based intervention for viral infections

**DOI:** 10.1002/btm2.10434

**Published:** 2022-11-15

**Authors:** Sherri Newmyer, Marvin A. Ssemadaali, Harikrishnan Radhakrishnan, Harold S. Javitz, Parijat Bhatnagar

**Affiliations:** ^1^ Biosciences Division SRI International Menlo Park California USA; ^2^ Education Division SRI International Menlo Park California USA

**Keywords:** cell engineering, cell therapies, electrogenetics, synthetic biology, viral infections

## Abstract

This work reports on an engineered cell that—when electrically stimulated—synthesizes a desired protein, that is, *ES‐Biofactory*. The platform has been used to express interferon (IFN)‐β as a universal antiviral protein. Compelling evidence indicates the inevitability of new pandemics and drives the need for a pan‐viral intervention that may be quickly deployed while more specific vaccines are in development. Toward this goal, a fast‐growing mammalian cell (*Chassis*) has been engineered with multiple synthetic elements. These include—(1) a voltage‐gated Ca^2+^ channel (*Voltage‐Sensor*) that, upon sensing the electric field, activates the (2) Ca^2+^‐mediated signaling pathway (*Actuator*) to upregulate (3) IFN‐β, via an engineered antiviral transgene (*Effector*), that is, *ES‐Biofactory*➔*IFN‐β*. The antiviral effects of the *ES‐Biofactory*➔*IFN‐β* have been validated on severe acute respiratory syndrome coronavirus 2 (SARS‐CoV‐2)‐infected cells. The irradiated *ES‐Biofactory*, that does not exhibit oncogenic capacity, continues to exert antiviral effect. The resulting *ES‐Biofactory*➔*IFN‐β* uses a novel signaling pathway that, unlike the natural IFN synthesis pathway, is not subject to viral interference. Once clinically validated, the *ES‐Biofactory* will be a universal antiviral cell therapy that can be immediately deployed in the event of an outbreak. The platform may also be useful in treating other diseases including cancer and autoimmune disorders.

## 1 INTRODUCTION

Estimates suggest that there are hundreds of thousands of zoonotic pathogens in wildlife reservoirs,^1^ and new viruses will continue to cross the species barrier into humans. Indeed, DNA results from people alive today suggest evidence of a coronavirus epidemic some ~25,000 years ago.^2^ Viral infections are responsible for the deadliest of pandemics in history, including the 1918 influenza epidemic (~50 million deaths), the HIV/AIDS epidemic (~35 million deaths to date),^3^ and the ongoing COVID‐19 pandemic (15 million deaths through 2022, as per WHO estimates,^4^ and counting). Climate change helps drive viral proliferation, and research indicates there will be more than 15,000 cases of zoonotic cross‐overs in the next 50 years.^5^ Consistent with this assessment and based on data from the past 400 years, a 3‐fold increase in the rate of disease emergence is expected in the coming decades and the probability of experiencing a pandemic in one's lifetime is ~38%.^6^ Clearly, the existing pandemic and future pandemics present an existential threat to all mankind. A universal treatment that can be rapidly implemented to treat viral outbreaks is needed and will stem loss of life while vaccines that elicit virus‐specific convalescent responses are developed.

Interferons (IFNs) serve as the first line of defense and clear most viruses during the incubation and prodromal stages of infection. They are synthesized as part of the IFN‐synthesis pathway that is activated when pattern recognition receptors are stimulated by virus‐related pathogen‐associated molecular patterns and virus‐induced damage‐associated molecular patterns.^7^, ^8^, ^9^ The resulting autocrine/paracrine signaling induces hundreds of IFN‐stimulated genes (ISGs) to suppress the infection. However, viruses continue to evolve and adapt to abrogate and evade the IFN‐synthesis pathway.^10^, ^11^ They also can induce amplified IFN production after the infection has been established that has been associated with the onset of severe disease.^12^, ^13^ IFN therapy that balances these antiviral and immunomodulatory roles has been used to successfully treat viral infections.^14^, ^15^, ^16^ However, the effectiveness of treatments for severe acute respiratory syndrome coronavirus 2 (SARS‐CoV‐2) has been mixed because the ideal timing, dosage, and route of administration are not known. In some cases, IFN therapy increases disease severity by causing complications such as inflammation, tissue damage, and multiorgan failure that has been attributed to the misalignment in dose regimen.^17^, ^18^ Notably, IFN dosing is negatively impacted by the size of the molecule, sensitivity to degradation, and rapid clearance.^19^, ^20^ As a result, new formulations for encapsulating IFN to enable its controlled release while limiting toxicity are in development.^21^ Ideally, these delivery systems would offer zero‐order release kinetics^22^ to provide a sustained and effective level of paracrine signaling to counter the spread of viral infection while preventing fluctuating IFN levels that are either toxic or ineffective. In this context, cell‐based therapies are evolving to both manufacture and deliver therapeutic proteins in vivo with sustained kinetics^23^, ^24^ and could potentially transform IFN therapy to effectively treat emerging viruses.

In a recent parallel study,^25^ we engineered a T‐cell line to synthesize different IFNs to trigger antiviral immunity via an alternative synthetic pathway that is not susceptible to viral interference, also known as *anti‐SARS T‐cell Biofactory*. We selected IFN‐β among different IFNs for its antiviral efficacy and demonstrated that, when delivered through the engineered cell, this cytokine stimulates ISG‐based universal antiviral effects.^25^ The anti‐SARS T‐cell Biofactory enabled in situ localized IFN delivery but required re‐engineering of the chimeric antigen receptor (CAR) that is integral to functioning of the downstream synthetic signaling pathway. The CAR molecule that binds spike envelope protein was used to target SARS‐CoV‐2. It can be reprogrammed for treating new viruses but this requires knowledge of the envelope protein of each new virus and the sequence of the targeting antibody^26^; determining these characteristics takes time and delays the public health response in an emerging outbreak. In this study, we describe a technology that obviates the need to define the virus's envelope protein, envelope‐specific single‐chain variable fragment sequence, virus‐specific culture conditions or reagents and demonstrate the possibility of a universal antiviral treatment that may be readily deployed during an outbreak. Toward this goal, we engineered the K562 cell line, a fast‐growing non‐excitable cell line^27^ (doubling time ~20 h), to activate upon electrical stimulation and synthesize therapeutic proteins. We *postulated* that using electrical stimulation, rather than antigenic stimulation,^25^, ^26^ could eliminate the need to reprogram the T‐cell Biofactory platform for each new virus and activate the non‐canonical synthetic IFN‐producing pathway that viruses have not evolved to hijack.

## 2 RESULTS

**FIGURE 1:**
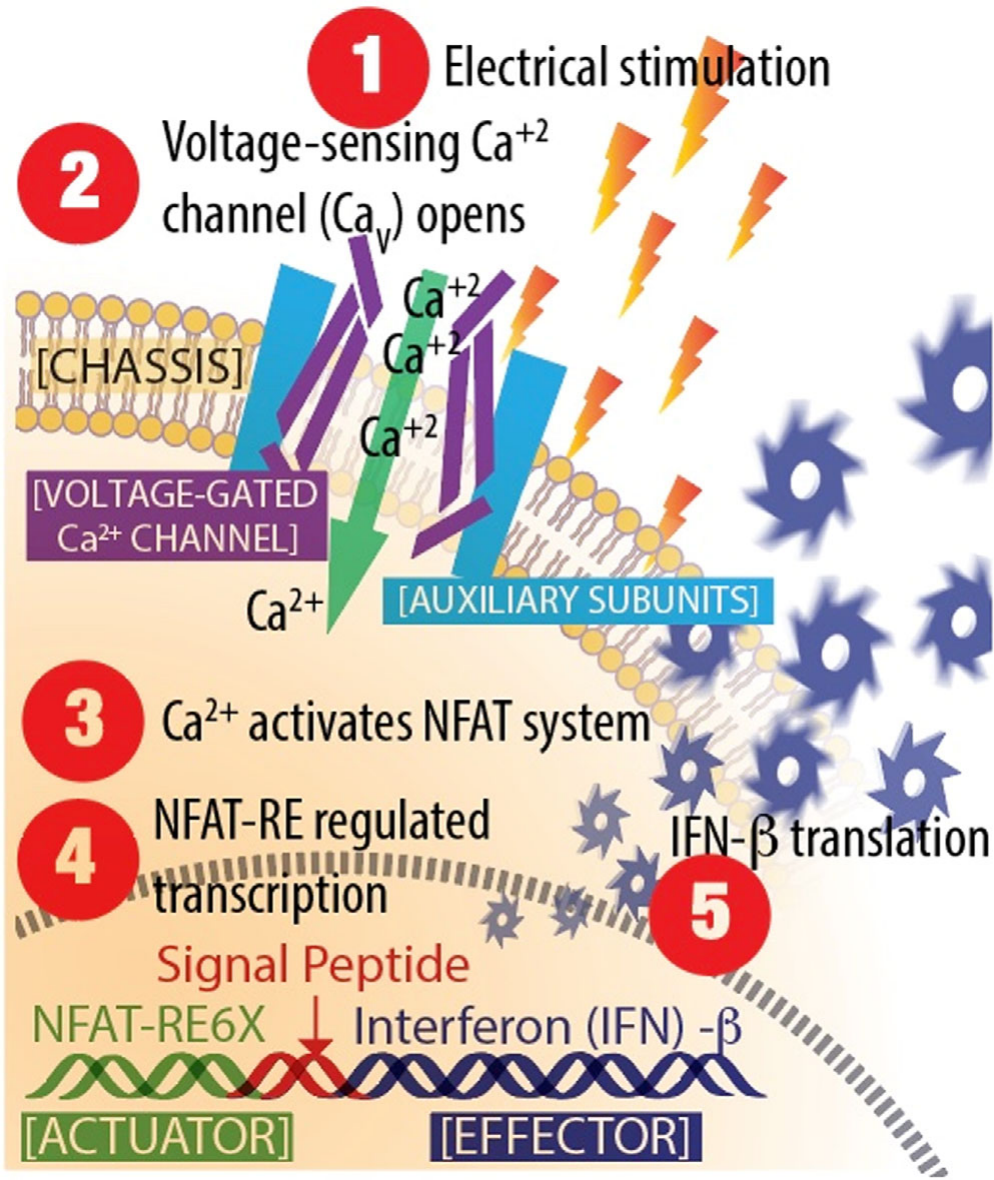
Schematic of ES‐Biofactory➔interferon (IFN)‐β function. (1) Electrical stimulation is applied to the ES‐Biofactory➔IFN‐β cell based in the K562 cell Chassis. (2) The Voltage‐Sensor, composed of the L‐type voltage‐gated Ca^2+^ channel Ca_V_1.2α_1_ subunit and α_2_δ_1_, and β_2_ auxiliary subunits, responds and causes a rise in intracellular Ca^2+^. (3) Ca^2+^ signaling induces nuclear translocation of the transcription factor NFAT. (4) Transcription is directed by the synthetic NFAT‐RE6X Actuator. (5) The signal peptide directs secretion of the newly synthesized Effector, IFN‐β

The schematic in Figure 1 shows the engineered cell with the *electrically responsive synthetic signaling pathway* to direct IFN‐β expression (ES‐Biofactory➔IFN‐β). A voltage‐sensing module (*Voltage‐Sensor*) composed of an L‐type voltage‐gated Ca^2+^ channel (Ca_V_α_1_) subunit (Ca_V_1.2 or Ca_V_1.3)^28^ and auxiliary subunits (α_2_δ_1_, β_2_, or β_3_) was engineered into a clinically relevant^29^, ^30^, ^31^ K562 cell (*Chassis*). The auxiliary subunits increase Ca^2+^ influx by regulating the opening and closing of the Ca_V_α_1_ channel^32^, ^33^ and by trafficking the channel to the cell surface.^34^, ^35^ The influx of Ca^2+^, triggered by electrical stimulation, acts as a second messenger system to initiate calcineurin‐mediated dephosphorylation of the nuclear factor of activated T‐cell (NFAT) transcription factors,^36^ which then traffic to the nucleus of the cell and interact with six copies of NFAT response element arranged in tandem (NFAT‐RE6X) (*Actuator*). This mobilizes the cell's transcriptional machinery to synthesize desired transgenic encoded proteins (*Effector*)—in this case, the type‐I IFN‐β known to suppress viral infection.^14^, ^15^, ^16^, ^37^, ^38^ We determined the therapeutic and prophylactic antiviral effects of IFN‐β produced by the ES‐Biofactory➔IFN‐β on SARS‐CoV‐2‐infected cells. To mitigate any potential oncogenesis‐related risk of ES‐Biofactory treatment, we explored the radiation dose (γ‐radiation) that renders the cell non‐proliferative^29^ without compromising its capacity to produce Effector IFN‐β. Ensuring safe administration of ES‐Biofactory➔IFN‐β manufactured in compliance with current Good Manufacturing Practices for Phase I/II clinical trials, therefore presents a translational approach that may be used as a universal antiviral against different pathogenic viruses.^39^, ^40^ Our rationale for developing this technology is that the universal antiviral cell therapy, once approved by regulatory agencies, may be readily deployed in pandemic situations to mitigate severe disease without any virus‐specific modification.

### 2.1 Investigation of electrical parameters for activating ES‐Biofactory

**FIGURE 2:**
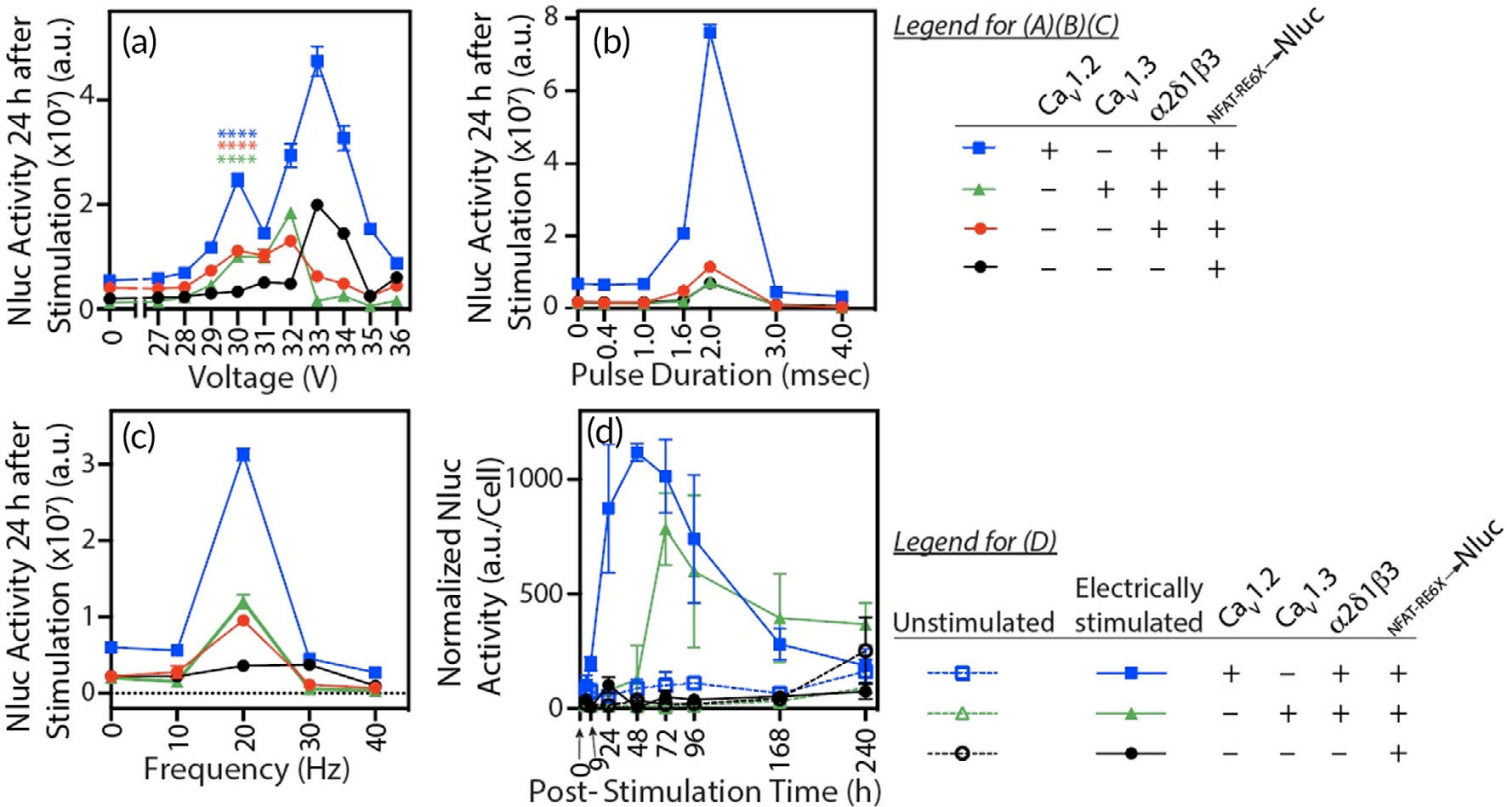
Investigation of electrical parameters that trigger ES‐Biofactory Effector expression. Following electrical stimulation, 16,500 of initially plated ES‐Biofactory➔Nluc cells (Chassis: Jurkat) were assayed for Effector (Nluc) activity. Composition of the Voltage‐Sensor module is indicated in the legend. Unless indicated on the *x* axis, the parameters included applied voltage = 30 V, frequency = 20 Hz, pulse duration = 2 ms, stimulus duration = 1 h, and post‐stimulus incubation = 24 h. A range of (a) voltage, (b) pulse duration, and (c) frequency‐enabled identification of the process window for optimally activating the ES‐Biofactory at 30 V. (d) The time course of Nluc response was normalized to the number of live cells and showed Effector expression increasing up to 48 h (Ca_V_1.2) and 72 h (Ca_V_1.3) after 1 h of stimulation. The cells were diluted 10‐fold following electrical stimulation to prevent cell density from limiting cell growth during the time course. Improved kinetics and increased Nluc activity in the ES‐Biofactory engineered with Ca_V_1.2, compared to Ca_V_1.3, identified Ca_V_1.2 as a promising Ca^2+^ channel to generate a more responsive ES‐Biofactory. Nluc activity for all observations was determined using *n* = 4, error bars indicate ±1 standard deviation (*SD*). For Panel A, the statistical significance of the difference of means (with unstimulated signal background subtracted) compare cell lines engineered with Voltage‐Sensor components and Actuator NFAT‐RE6X➔Nluc (see legend) against the cell line engineered with only NFAT‐RE6X➔Nluc was determined with the unpaired two‐sample *t*‐test (Ca_V_1.2^+^Ca_V_1.3^neg^α_2_δ_1_β_3_^+^, Ca_V_1.2^neg^Ca_V_1.3^+^α_2_δ_1_β_3_^+^, and Ca_V_1.2^neg^Ca_V_1.3^neg^α_2_δ_1_β_3_^+^ vs. NFAT‐RE6X➔Nluc alone, *p* < 0.0001, *p* = 0.0003, and *p* < 0.0001, respectively).

The two voltage‐gated Ca^2+^ channels (Ca_V_α_1_ pore), Ca_V_1.2, and Ca_V_1.3, were initially investigated as part of the Voltage‐Sensor by replacing the antigen‐specific CAR described in our previous publications on the *Cell Biofactory* technology^26^, ^41^, ^42^, ^43^ using the Jurkat cell line Chassis. In response to an electrical stimulus and in combination with auxiliary subunits (α_2_δ_1_, β_3_) that mediate Ca^2+^ signaling in lymphocytes,^44^ both channels activated Effector transgene expression. We used NanoLuc® (Nluc), a bioluminescent reporter enzyme, as a surrogate Effector protein, that is, ES‐Biofactory➔Nluc to enable accurate investigation of the parameters and optimally stimulate the ES‐Biofactory; the Nluc sequence can be exchanged with a therapeutic Effector such as IFN‐β. The results are shown in Figure 2 and differentiate the performance of the ES‐Biofactory using Ca_V_1.2 or Ca_V_1.3 with auxiliary subunits as components of the Voltage‐Sensor module. Figure 2a shows representative data for Nluc Effector activity with the *x* axis depicting the applied voltage at a resolution of one volt. The Effector expression increased with rising voltage between 29 and 33 V, and the trend was comparable to that observed with voltage‐dependent gating of Ca_V_1.2 and Ca_V_1.3 channels.^45^ The Effector expression declined beyond ~33 V of applied voltage (Figure 2a), and cell viability decreased with increasing strength of the electrical stimulus (Figures S1A,B). The *x* axis in Figure S1C shows Nluc Effector activity in ES Biofactory➔Nluc and depicts the applied voltage at a lower resolution of 5 V as compared to a chemically stimulated positive control. A 60‐min application of 30 V at 20 Hz frequency and 2 ms pulse duration was an optimal stimulus to maximize electrical response and preserve cell viability (Figure S2A,B respectively). With electrical stimulation, all cells engineered with different Voltage‐Sensor components (i.e., Ca_V_1.2^+^α_2_δ_1_β_3_^+^, Ca_V_1.3^+^α_2_δ_1_β_3_^+^, Ca_V_1.2^neg^Ca_V_1.3^neg^α_2_δ_1_β_3_^+^) expressed higher Effector activity compared to negative control cells engineered with only the *Actuator‐Effector* module (i.e., Ca_V_1.2^neg^Ca_V_1.3^neg^α_2_δ_1_β_3_^neg^) (Figure 2a [*p* < 0.0001 at 30 V] and Figure S2C). The electrical activation of the ES‐Biofactory to synthesize the Effector protein is a function of applied electric field (defined as applied voltage ÷ electrode spacing) rather than applied voltage. We confirmed this by comparing the voltage needed to trigger the cells when placed between two differently spaced electrodes. Our experiment showed that ~2.5× higher voltage is needed for 28‐mm electrode spacings (6‐well plate) than 12‐mm spacings (24‐well plate) (Figure S2D,E). The studies we describe below used the 6‐well format to provide adequate cell numbers for post‐stimulus work‐up.

An iteratively conducted set of experiments informed on the range of different process parameters that electrically induce robust Effector expression (Figure 2b–d). Representative data demonstrating the relationship of pulse duration and frequency to drive Effector synthesis are shown in Figure 2b,c, respectively. At 30 V, a pulse duration of 2 ms and frequency of 20 Hz triggered the ES‐Biofactory. However, the voltage required to stimulate the ES‐Biofactory for Effector expression decreased with increasing stimulation frequency. For example, the ES‐Biofactory treated at 20 V with a pulse duration of 2 ms required a frequency of 45 Hz to activate Effector expression (contrast Figure 2c with Figure S3). We compared the time course of Effector expression of the two differently configured ES‐Biofactory models (i.e., Ca_V_1.2^+^α_2_δ_1_β_3_^+^ and Ca_V_1.3^+^α_2_δ_1_β_3_^+^) after stimulating them for 1 h. The kinetics and magnitude of Effector expression were faster and greater in the Ca_V_1.2^+^α_2_δ_1_β_3_^+^ES‐Biofactory compared to the Ca_V_1.3^+^α_2_δ_1_β_3_^+^ES‐Biofactory (Figure 2d) when normalized to the number of live cells (see Figure S4 for non‐normalized data).

Cumulatively, the results presented in Figure 2 show both ES‐Biofactory (Ca_V_1.2^+^α_2_δ_1_β_3_^+^ and Ca_V_1.3^+^α_2_δ_1_β_3_^+^) are functional. However, the CaV1.2‐equipped ES‐Biofactory responds faster and with greater magnitude. These factors were used to justify the use of Ca_V_1.2 over Ca_V_1.3, and Ca_V_1.2 was co‐expressed with α_2_δ_1_β_3_ to compose a fully functional Voltage‐Sensor module. The two negative controls (i.e., *Actuator*‐*induced Effector* expression with and without the auxiliary subunits but without the Ca_V_α_1_ subunit, i.e., Ca_V_1.2^neg^Ca_V_1.3^neg^α_2_δ_1_β_3_^+^ and Ca_V_1.2^neg^Ca_V_1.3^neg^α_2_δ_1_β_3_^neg^) also exhibited background Effector activity. We did not investigate the cause but suspect endogenous Ca_V_ expression in T cells^44^ may contribute, albeit weakly, to voltage sensing.

### 2.2 Investigation of biological parameters for activating ES‐Biofactory

**FIGURE 3:**
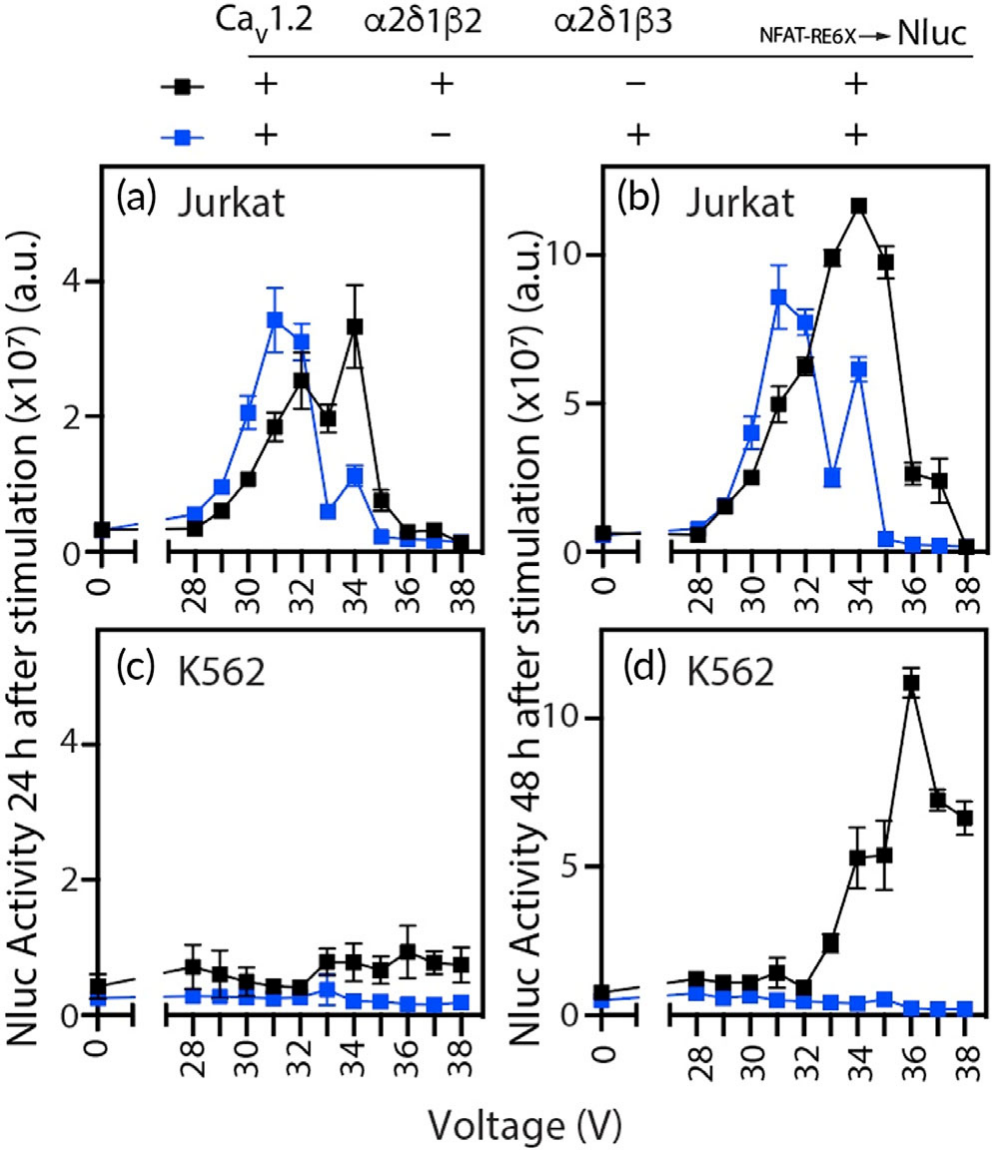
Investigation of biological parameters that drive ES‐Biofactory Effector expression. Following electrical stimulation, 16,500 of initially plated ES‐Biofactory➔Nluc cells were assayed for Effector (Nluc) activity. Electrical stimulus comprised applied voltage = 0–38 V, frequency = 20 Hz, pulse duration = 2 ms, and stimulation duration = 1 h. Effector (Nluc) activity was measured 24 h (a,c) and 48 h (b,d) after initiation of electrical stimulus and analyzed as a function of auxiliary β subunit (β_2_, β_3_) in two different cellular Chassis: (a,b) Jurkat and (c,d) K562. In both cell lines, the ES‐Biofactory platform included α_2_δ_1_ as additional auxiliary subunits and Ca_V_1.2 as the Ca^2+^ channel component of the Voltage‐Sensor module. Both β subunits promoted reporter expression in the Jurkat cell‐based ES‐Biofactory. Only β_2_, but not β_3_, supported reporter expression in the K562 cell‐based ES‐Biofactory. Nluc activity for all observations was determined using *n* = 4, error bars indicate ±1 *SD*.

We explored biological parameters (i.e., auxiliary subunit expression and the cell Chassis) to determine regulation of the Voltage‐Sensor by the β isoform and the applicability of the synthetic signaling pathway to function in the clinically relevant K562 cell line (Figure 3). We tested two different β isoforms (β_2_ and β_3_) as part of the auxiliary subunit component of the Voltage‐Sensor in the Jurkat and K562 Chassis in combination with Ca_V_1.2. We encoded the β_2_ subunit, and specifically the β_2a_ splice variant, as part of the Voltage‐Sensor to learn how this isoform might better enable Ca^2+^ influx through the Voltage‐Sensor by improving trafficking to the cell surface and slowing inactivation of the Ca_V_α_1_ pore.^46^, ^47^ Within the Jurkat cell Chassis, β_3_ and β_2_ supported electrical triggering of the Voltage‐Sensor to induce Effector expression. This was detected at 24 h (Figure 3a) and continued to increase through 48 h (Figure 3b). In contrast, only the β_2_ subunit induced Effector expression in K562 cells. Although undetected at 24 h (Figure 3c), it increased to similar levels as in the Jurkat cells at 48 h (Figure 3d). The β_2_ subunit may better support signaling by the Voltage‐Sensor in K562 cells by increasing the trafficking of Ca_V_1.2 to the plasma membrane,^47^, ^48^ which then enhances influx of the second messenger Ca^2+^ to drive Actuator‐induced Effector expression. In summary, these studies demonstrate that Effector expression can be electrically induced by the Voltage‐Sensor module composed of Ca_V_1.2^+^α_2_δ_1_β_3_^+^ in Jurkat cells and of Ca_V_1.2^+^α_2_δ_1_β_2_^+^ in both Jurkat and K562 cells.

We have previously developed an antigen‐sensing *T‐cell Biofactory* ^42^ and a natural killer (*NK‐cell Biofactory*)^43^ that, upon engaging the target antigen,^49^ activate the transcriptional machinery to mobilize the Actuator and respond by expressing an Effector. However, artificially orchestrating this signaling through antigenic stimulation was not successful with the K562 Chassis.^50^ This might be due to missing co‐stimulatory molecules in K562 cells that are necessary for activation of immune cells.^49^ By introducing the appropriate Voltage‐Sensor in this study, we circumvented the classical co‐stimulatory signaling pathway that is missing in non‐excitable K562 cells. This strategy allowed us to activate engineered K562 cells for regulated in situ Effector synthesis and to lay the groundwork for directing the K562‐based ES‐Biofactory platform against SARS‐CoV‐2.

### 2.3 Investigation of antiviral function of the ES‐Biofactory➔IFN‐β

**FIGURE 4:**
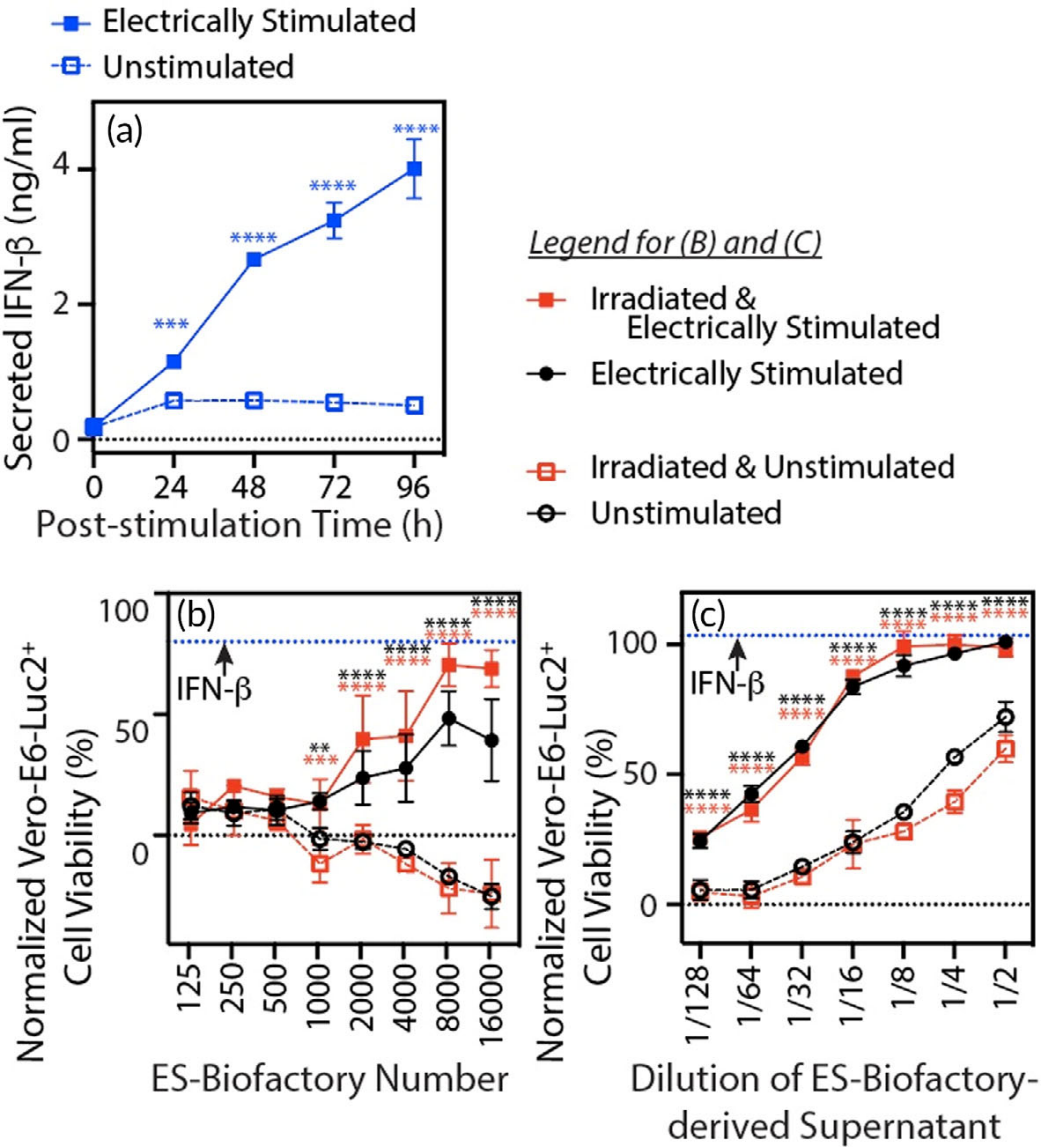
K562‐based ES‐Biofactory driven interferon (IFN)‐β secretion suppresses SARS‐CoV‐2‐mediated host cell killing. Following electrical stimulation, ES‐Biofactory➔IFN‐β cells (Chassis: K562) were assessed for (a) Effector (IFN‐β) production, (b) therapeutic activity, and (c) prophylactic activity. Electrical stimulus comprised applied voltage = 34 V, frequency = 20 Hz, pulse duration = 2 ms, and stimulation duration = 1 h. (a) A time course of Effector (IFN‐β) production was measured by harvesting the supernatant from the ES‐Biofactory culture at the designated time point after initiation of electrical stimulus and quantifying IFN‐β signaling. In response to electrical stimulation, the ES‐Biofactory produced IFN‐β that increased over the course of 96 h. The antiviral (b) therapeutic and (c) prophylactic activity of the ES‐Biofactory‐secreted IFN‐β was assessed by measuring cell viability in Vero‐E6‐Luc2^+^ host cells 48 h after SARS‐CoV‐2 infection and was compared with 0.1 μg (1 µg/ml) of purified IFN‐β as a positive control. Cell viability was normalized with 100% representing the luminescence value measured for the untreated, healthy Vero‐E6‐Luc2^+^ cells and 0% representing that for the SARS‐CoV‐2‐infected cells. In a therapy treatment protocol (b), the ES‐Biofactory➔IFN‐β cells were electrically stimulated and co‐cultured with Vero‐E6‐Luc2^+^ cells infected with SARS‐CoV‐2 prior to the co‐culture. In a prophylaxis treatment protocol (c), IFN‐β containing supernatant was harvested from ES‐Biofactory➔IFN‐β cells 48 h after electrical stimulation. Vero‐E6‐Luc2^+^ cells were pretreated with serially diluted IFN‐β supernatant prior to SARS‐CoV‐2 infection. In both protocols, a statistically significant antiviral effect was observed with the electrically stimulated ES‐Biofactory➔IFN‐β treatment compared to their non‐stimulated negative controls. The irradiated ES‐Biofactory➔IFN‐β also retained the ability to suppress virus‐mediated cell killing. IFN‐β production and cell viability for all observations were determined using *n* = 3, error bars indicate ±1 *SD*. In all panels, the indicated statistical significance of the difference of means was determined with the unpaired two‐sample t test using a common variance; only differences that were statistically significant at the Benjamini, Krieger, and Yekutieli false discovery rate of 0.01 are reported.

The surrogate Effector of the ES‐Biofactory➔Nluc configured with optimal Voltage‐Sensor and Actuator domains, as described above, was replaced with the IFN‐β1 transgene and engineered into the K562 cell Chassis (Figure 4). We used the resulting ES‐Biofactory➔IFN‐β to assess and validate application of the ES‐Biofactory platform for regulated synthesis of desired biologic therapeutics. IFN‐β protein, synthesized by the ES‐Biofactory➔IFN‐β upon electrical stimulation, was detected in the supernatant at 24 h and increased through 96 h (Figure 4a, *p* < 0.0002 for all time points). Using protocols designed to simulate therapeutic (Figure 4b) and prophylactic (Figure 4c) effects, we subsequently analyzed the antiviral function of IFN‐β secreted by the electrically stimulated ES‐Biofactory on SARS‐CoV‐2 infection of Vero‐E6‐Luc2^+^ cells. To simulate therapeutic application of the ES‐Biofactory, we co‐cultured the electrically stimulated ES‐Biofactory➔IFN‐β cell with Vero‐E6‐Luc2^+^ cells immediately following their infection with SARS‐CoV‐2 (Figure 4b). The electrically stimulated ES‐Biofactory protected the Vero‐E6‐Luc2^+^ host cells against viral replication as compared to the unstimulated ES‐Biofactory (*p* < 0.0002 at ES‐Biofactory➔IFN‐β cell counts greater than 2000). Antiviral activity improved with increasing number of ES‐Biofactory cells. To assess prophylactic antiviral activity, we pretreated the Vero‐E6‐Luc2^+^ cells with the supernatant of electrically stimulated ES‐Biofactory➔IFN‐β prior to viral infection. In this experiment, preemptive exposure to IFN‐β‐containing supernatant harvested from the electrically activated ES‐Biofactory➔IFN‐β significantly suppressed the cytopathic effect in the infected host cell population compared to a similar application of the supernatant from the unstimulated ES‐Biofactory➔IFN‐β (Figure 4c, *p* < 0.00001 at all supernatant dilutions).

Additionally, we determined the effect of irradiating cells to eliminate potential oncogenesis and qualify the cell‐based therapies for human use.^29^, ^51^ Our data showed that irradiating the K562‐based ES‐Biofactory➔IFN‐β at 30 Gy was sufficient to abrogate their proliferative capacity (Figure S5A) while maintaining cell Effector function (Figure S5B). Therapeutic and prophylactic antiviral functions (Figure 4b,c) were preserved when the ES‐Biofactory➔IFN‐β was irradiated prior to electrical stimulation (stimulated versus unstimulated, *p* < 0.01 at ES‐Biofactory➔IFN‐β cell counts greater than 1000 for therapeutic effect; *p* < 0.00001 at all supernatant dilutions for prophylactic effect), thereby substantiating the potential for translating the K562‐based ES‐Biofactory platform for use in clinical applications.

In parallel efforts guided by the results in Figures 2 and 3 with the Jurkat‐based ES‐Biofactory➔Nluc, we also investigated IFN‐β production by Jurkat‐based ES‐Biofactory➔IFN‐β. Applying the electrically responsive synthetic signaling pathway in irradiated and non‐irradiated Jurkat cells for controlled synthesis of IFN‐β also demonstrated therapeutic and prophylactic trends (Figure S6). Note that therapeutic treatment with irradiated cells seemed to suppress viral infection more effectively in Figure 4b and Figure S6B than with unirradiated cells. Although we did not conduct systematic investigations of this phenomena, antiviral efficacy among repeated experiments correlated with total IFN production, as assayed in parallel, and not irradiation treatment. This could be explained by irradiation induced IFN production^52^ or increased regenerative potential.^53^ Alternatively, it is possible that compounding variability in biological response to two processes, electrical stimulation and irradiation, caused heterogeneity in induced level of Effector protein expression. For example, our data already indicated variability in Nluc expression from the ES‐Biofactory➔Nluc in response to electrical stimulation (Figure S2A). Regardless, the antiviral effects manifested by the ES‐Biofactory with both cell Chassis, Jurkat and K562 cell lines, demonstrated proof of concept that the platform can be used in multiple cell types to synthesize desired therapeutic proteins.

## 3 DISCUSSION

There is no perfect way to implement an effective public health response during a pandemic. Each virus manifests in a different manner, produces varied clinical symptoms, and possesses a unique molecular structure. Vaccines prime adaptive humoral and cell‐based immunity to directly act on invading pathogens. Nevertheless, developing a vaccine is a time‐ and resource‐intensive process for each pathogen.^54^ For example, preparing a vaccine to counter SARS‐CoV‐2 took more than a year, and many lives were lost during that time. New variants emerged that escaped vaccine‐induced immunity and triggered new waves of illness that further threatened public health and the world economy. A treatment that can be quickly deployed to treat severe illness while pathogen‐specific vaccines are in development is critically needed.

A universal intervention that exerts pan‐viral efficacy to mitigate severe disease, once approved by the U.S. Food and Drug Administration, can be immediately deployed in an outbreak. The ES‐Biofactory➔IFN‐β developed in this work demonstrates proof of concept that a K562 cell line, a clinically relevant cell Chassis, can be electrically triggered to provide IFN‐based innate immunity against pathogenic viruses without establishing their molecular identity. This offers a new paradigm to regulate IFN bioavailability and effectively suppress the clinical progression of viral disease to severe stages.

We were able to synthesize IFN‐β and achieve the desired antiviral effect using an irradiated K562‐based ES‐Biofactory➔IFN‐β cell line. Such irradiation eliminates potential oncogenesis and improves the safety of this cell‐based intervention. The use of K562 cells is advantageous because it further increases safety of the therapy. This is because these cells lack the major histocompatibility complex molecules and in the event of potential oncogenesis can be cleared by NK cells.^55^ Following development and regulatory approval, the ES‐Biofactory➔IFN‐β will be available as a universal therapeutic to buy time for more specific direct‐acting prophylactic and therapeutic interventions to be developed. The fast‐growing K562 cell Chassis is easy to deploy because it can be distributed in small quantities across the globe and rapidly scaled on site. The ES‐Biofactory will, therefore, have rapid and broad applicability in any viral outbreak.

Unlike the innate IFN synthesis pathway that is often dysregulated in viral diseases,^10^, ^11^, ^56^ we have introduced an alternative route for enabling IFN‐β bioavailability via an electrically responsive engineered cell. The innovation in the ES‐Biofactory➔IFN‐β is the introduction of a non‐natural, voltage‐sensitive, synthetic signaling pathway that is not predisposed to viral interference. It decouples IFN synthesis from the innate pathways to regulate its production upon stimulation by electric field. As such, in the event of an outbreak, the use of ES‐Biofactory➔IFN‐β as a therapeutic against a virus will be advantageous as it eliminates the need to identify the viral molecular structure while IFN‐β synthesis can be regulated via an extracorporeal device.

Other approaches have been used to deliver IFN therapies, including subcutaneous or intramuscular injection using first‐order drug delivery systems, for example, liposomes, pegylation, hydrogels, microparticles, and so forth, that may reduce immunogenicity and enhance bioavailability.^21^, ^24^ Nevertheless, challenges continue to exist. For example, mammalian cell and bacterially expressed formulation of IFN‐β therapies approved for clinical use, IFN‐β1a and IFN‐β1b, cause systemic toxicities ranging from flu‐like symptoms, myalgia to leukopenia and liver damage.^57^, ^58^ In addition, IFN‐β1b lacks appropriate post‐translational modification,^59^ and repeated dosing is required to compensate for proteolysis and renal clearance.^19^, ^20^ Pegylation of IFN‐β1a improves maintenance of effective plasma concentration of the protein, increasing the elimination half‐life from 12 h in the non‐pegylated formulation to 72 h when administered subcutaneously.^60^ However, pegylation decreases antiviral IFN activity to 50% for IFN‐β1a, 7% for IFNα2a and 28% for IFNα2b when compared to the parental proteins^61^, ^62^; and has been reported to cause nerve degeneration,^63^ renal tubular vacuolation,^64^ complement activation,^65^ and general immunogenic responses via anti‐polyethylene glycol (PEG) antibodies.^66^ The subcutaneous administration of pegylated IFN‐β1a still exhibits uncontrolled release kinetics including an initial burst release that peaks and requires weekly dosing to maintain effective dosing.^60^ In contrast, the ES‐Biofactory offers a zero‐order drug delivery system.^67^ It will enable sustained in situ production of IFN‐β that, unlike direct administration of protein, will not require multiple infusions and will not cause adverse fluctuating systemic concentrations. With the first approved use of IFN‐β in 1993, extensive research on this cytokine has improved its production and bioavailability and new formulations aim to extend the delivery window.^68^ Building on this IFN work, the ES‐Biofactory➔IFN‐β offers the opportunity to tune in situ IFN production to meet disease and patient need and turn production off to improve the safety of IFN treatment and reduce side effects.

Given that IFN therapy administered to COVID‐19 patients shortly after onset of symptoms prevented disease progression to severe stages, shortened hospital stays, and reduced viral load, we expect early‐stage use of the ES‐Biofactory➔IFN‐β will prompt a similarly effective response. Notably, a successful antiviral countermeasure will benefit vulnerable populations, that is, the elderly and those with comorbidities, for whom the only option has been social isolation, which negatively affect mental and physical health. We selected SARS‐CoV‐2 as a model to represent emerging viruses; however, the impact of this work will be far‐reaching because IFN‐β also has therapeutic applications in other diseases including bacterial infections, autoimmune disorders, and solid tumors.^69^, ^70^ The modularity of the ES‐Biofactory enables customization of the Effector protein for different genetic payloads and use of a different cell Chassis to address various therapeutic needs. For example, in vivo regulation of an insulin‐producing human islet‐derived β‐cell line with an electrically responsive trigger housed in an implanted bioelectronic device has been demonstrated.^71^ A porous implant would enable both IFN efflux into the blood stream and the diffusion of nutrients and oxygen to maintain the encapsulated ES‐Biofactory cells while protecting them from attack by the immune system. Such an implant, equipped with wireless electrodes, has been used to electrically activate cells for systemic insulin delivery in a mouse model.^71^ However, additional effort is needed to translate a similar device for human use, including scaling the cell number to meet therapeutic need.^72^ The ES‐Biofactory➔IFN‐β reported in this work expands the capability of electrically‐responsive therapeutic cells and highlights mechanisms to improve its performance. For example, determining how the Jurkat cell Chassis can rapidly express therapeutic Effectors may further guide efforts to improve transcriptional responsiveness of the ES‐Biofactory. In the continuum of this study, we envision a cellular system with a binary on–off switch for regulated in situ synthesis of therapeutic proteins of human or non‐human origin or robust recombinant versions.

## 4 MATERIALS AND METHODS

### 4.1 Materials and reagents

Engineered Jurkat E6‐1 (ATCC, Cat #TIB‐152) and K562 (ATCC, Cat #MCCL‐243) cell lines were maintained in complete Roswell Park Memorial Institute (RPMI) media (RPMI1640 [Corning, Cat #10‐041‐CV], 10% heat‐inactivated fetal bovine serum [FBS] [Sigma‐Aldrich, Cat #F2442‐500Ml], and 1× Penicillin–Streptomycin solution [Corning, Cat #30‐002‐Cl]). Engineered Vero‐E6 cells (ATCC, Cat # CRL‐1586) were cultured in complete Eagle's Minimum Essential Medium (EMEM) (EMEM [Corning, Cat #10‐009‐CV], 10% heat‐inactivated FBS and 1× Penicillin–Streptomycin solution). All cells were expanded, and liquid nitrogen stocks were maintained using freezing media (50% FBS, 40% RPMI, and 10% dimethyl sulfoxide [DMSO]).

Plasmids encoding different genetic payloads (transfer plasmids) were designed in SnapGene software (GSL Biotech LLC) and sub‐cloned into piggyBac vector plasmid (System Biosciences, Cat #CD510B‐1) or piggyBac Transposon vector plasmid (System Biosciences, Cat # PB510B‐1). PiggyBac Transposase sequence was provided by Dr. Nancy Craig at the Johns Hopkins University School of Medicine.^73^ An insert for “EF1alpha promoter – i7pB transgene – bGH poly(A) signal” was chemically synthesized and assembled using overlapping polymerase chain reaction (PCR) products into pUC19 (GenBank: L09137, New England Biolabs, #N3041). All plasmid preparation services (chemical synthesis of DNA insert sequences, sub‐cloning into respective vector backbones, and the amplification) were obtained from Epoch Life Science, Inc. (Missouri City, TX).

Jurkat and K562 cell line engineering used the respective nucleofection kits: SE Cell line 4D‐Nucleofector™ X Kit S (Lonza Cat# V4XC‐1032) and SF Cell line 4D‐Nucleofector™ X Kit L (Lonza Cat# V4XC‐2024), according to manufacturers' instructions. Puromycin dihydrochloride (ThermoFisher Scientific, Cat# A1113803) and G418 Sulfate (Corning, Cat# 30‐234‐CI) were used to select stable cells. Nano‐Glo® Luciferase Assay (Promega #N1150) was used to measure Nluc activity. Cell viability was either determined with CellTiter‐Glo® 2.0 (Promega # G9242) or ViaStain™ AO/PI Cell Staining (Nexcelom Cat# CS2‐0106). Vero‐E6‐Luc2^+^ cells were engineered as described and Luc2^+^ used as a reporter of cell killing by SARS‐CoV‐2.^25^ Dr. Mary Lanier at SRI International provided the SARS‐CoV‐2 virus culture (BEI Resources, NIH; Hong Kong/VM20001061/2020 [Cat# NR‐52282]).

The viability of Vero‐E6‐Luc2^+^ cells was assessed using either the CellTiter‐Glo® 2.0 Cell Viability Assay (Promega, Cat# PR‐G7570) or the One‐Glo® assay (Promega, Cat #E6110). Human embyonic kidney (HEK)‐Blue IFN‐α/β cells (InvivoGen, Cat# hkb‐ifnab) were used to quantify IFN amount produced by the ES‐Biofactory➔IFN‐β, following manufacturer's instructions, in complete DMEM media (DMEM [Corning, Cat #10‐013‐CV], 10% heat‐inactivated FBS, and 1× penicillin–streptomycin solution). Recombinant human IFN‐β1a protein from R&D systems (Cat# 8499‐IF‐010/CF) was used as a control.

### 4.2 Generation of ES‐Biofactory (ES‐Biofactory➔Nluc; ES‐Biofactory➔IFN‐β)

Jurkat and K562 suspension cell lines were engineered with the piggyBac Transposon system as previously described.^74^ Transfer plasmids contained the following genetic components (Appendix A): Ca_V_1.2: Q13936‐20 (UniProt); Ca_V_1.3: Q01668‐1 (UniProt); α_2_δ_1_: P54289‐2 (UniProt); β_3_: P54284‐1 (UniProt); β_2a_: Q08289‐2 (UniProt); Nluc: JQ437370.1 (GenBank), IFN‐β1: P01574‐1 (UniProt). Following nucleofection with the transfer plasmid and the piggyBac Transposon vector, the transfected cells were cultured for 48 h and then placed in selection using 0.5 μg/ml of puromycin dihydrochloride or 1 mg/ml G418. Following selection, cells were expanded as required for different assays and frozen using freezing media. The ES‐Biofactory➔Nluc and ES‐Biofactory➔IFN‐β required two rounds of nucleofection to introduce Plasmid (A) Ca_V_1.2 or Ca_V_1.3 along with the Actuator and Effector modules (puromycin‐selected) and Plasmid (B) the α_2_δ_1_ and β auxiliary subunits (G‐418‐selected). See Appendix A (Supporting Information) for additional details.

### 4.3 Generation of irradiated ES‐Biofactory

The ES‐Biofactory was irradiated for the desired dose using a ^137^Cs γ‐emitting irradiator, Mark I‐68A (JL Shepherd and Associates) at a dose rate of 222 mGy/min. The control cells were treated similarly except for the irradiation.

### 4.4 Nluc reporter assay

Effector protein expression was measured with the Nano‐Glo® assay (Promega, Cat #N1120) according to manufacturer's instructions. Briefly, the Nluc substrate was diluted in cell lysis buffer and added 1:1 to treated cells transferred to a 96‐well plate. Bioluminescence was read on a microplate reader (Perkin Elmer, EnVision™ Multilabel Plate Reader, Model: 2104‐ 0010A).

### 4.5 IFN‐β quantification assay

IFN‐β production was determined using HEK‐Blue IFN‐α/β reporter cells. Secreted IFN was harvested by collecting the supernatant following centrifugation of the treated cells at 500× *g* for 5 min. Following manufacturers' instructions, 50 μl of the serially diluted supernatant was mixed with 150 μl 50,000 HEK‐Blue IFN‐α/β reporter cells in complete DMEM growth media and plated in a single well of a 96‐well plate. Recombinant IFN‐β1a, serially diluted (10‐fold) in DMEM growth media, was plated in parallel to generate a standard curve for IFN concentration. After 24 h of incubation, 20 μl of HEK‐Blue IFN‐α/β (or HEK‐Blue IFN‐λ) supernatant was added to 180 μl of Quanti‐blue substrate (InvivoGen) and incubated at 37°C. Absorbance was measured at 650 nm using a microplate reader (Perkin Elmer, EnVision™ Multilabel Plate Reader).

### 4.6 Electric field stimulation of ES‐Biofactory

Cells were cultured in either 6‐well or 24‐well dishes fitted with C‐Dish™ lids (IonOptix Cat# CLD6WFC and CLD24WF) to immerse two carbon electrode elements within each well. The cells were electrically stimulated with a biphasic electric field generated using the IonOptix C‐Pace EM stimulator, and the 6‐well format was used unless indicated otherwise.

### 4.7 Functional assessment of the ES‐Biofactory➔Nluc

In a typical experiment, 3 ml of cells were plated per well at the density of 0.17 × 10^6^ cells/ml. Exactly 24 h after plating, the cells were electrically stimulated for 1 h and then assayed for Effector protein expression 24–48 h after the onset of stimulus using the Nluc reporter assay described above.

### 4.8 Functional assessment of the ES‐Biofactory➔IFN‐β

Exactly 24 h before electrical stimulation, ES‐Biofactory➔IFN‐β cells were seeded at 3.3 × 10^5^ cells/ml in complete RPMI media. Prior to stimulation, the cells were divided into two sets and either left untreated or irradiated (Irradiation dose – Jurkat: 20 Gy, K652: 30 Gy). The irradiated and non‐irradiated cells were electrically stimulated for 1 h (Jurkat: 31 V, K562: 34 V). Frequency of 20 Hz and pulse duration of 2 ms was used in all cases. Prophylactic or therapeutic activity was assessed as described below.

#### 4.8.1 Assessing prophylactic effect

Exactly 48 h after stimulus initiation, the supernatant was harvested with 500× *g* centrifugation for 5 min and serially diluted (two‐fold) with complete EMEM media. Total 100 μl of serially diluted cell supernatants were then used to pre‐treat a monolayer of Vero‐E6‐Luc2^+^ cells for 24 h (20,000/well in a 96‐well plate, triplicates). Recombinant human IFN‐β1a (1 μg/ml) was included as a positive control. The pre‐treated Vero‐E6‐Luc2^+^ cells were then infected with SARS‐CoV‐2 virus culture in complete EMEM media at a multiplicity of infection (MOI) of 0.1 for 48 h. Cell viability was determined using the CellTiter‐Glo® 2.0 Cell Viability Assay following the manufacturer's instructions.

#### 4.8.2 Assessing therapeutic effect

Concomitant with electrical stimulation, a monolayer of Vero‐E6‐Luc2^+^ (20,000/well in a 96‐well plate, triplicates) was infected with SARS‐CoV‐2 virus culture at an MOI of 0.1 and incubated for 2 h to allow virus attachment. The virus inoculum was then removed and 100 μl of serially diluted ES‐Biofactory➔IFN‐β cells (two‐fold) were plated accordingly. Recombinant human IFN‐β1a (1 μg/ml) and non‐infected Vero‐E6‐Luc2^+^ cells were used as controls. After 48 h of co‐culture, Vero‐E6‐Luc2^+^ cell viability was determined by assessing Luc2 activity using the One‐Glo® assay kit, following manufacturer's instructions.

### 4.9 Statistical analysis

GraphPad Prism 9.2.0 (GraphPad Software, Inc.) was used to conduct all quantification and statistical analysis. For dot plots, the error bars extend 1 *SD* above and below the mean. All *p* values were calculated with the unpaired two‐sample *t*‐test with common variance. To adjust for multiple comparisons the two‐stage linear step‐up procedure of Benjamini, Krieger, and Yekutieli was used with a false discovery rate (FDR) of 1%; only statistical findings with FDRs (*q*‐values) less than 0.01 are reported. Statistical significance of the *p*‐values is indicated by asterisks: *p < 0.1, **p < 0.01, ***p < 0.001, and ****p < 0.0001. All reported confidence intervals are at the 95% level. For the box plots drawn in Figure S2C, the boxplot boundaries represent the 25th–75th percentiles. The line in the middle of the box is plotted at the median and the whiskers are drawn using the Tukey method.

Effector Nluc expression plotted in Figure 2d was normalized to viable cell count to show Nluc activity dependent on ES‐Biofactory activation and independent of cell count. The viable cell count was calculated by using a standard curve of known cell number values to scale the CellTiter‐Glo signal. Normalized Nluc activity = Nluc activity (from Figure S4A)/viable cell count (Figure S4B).

Cell viability in Figures 4b,c and S6B,C was normalized in GraphPad Prism with 100% representing the luminescence signal measured for the untreated, healthy Vero‐E6‐Luc2^+^ population and 0% representing that for the SARS‐CoV‐2‐infected cells.

## AUTHOR CONTRIBUTIONS

**Sherri Newmyer:** Formal analysis (lead); investigation (lead); methodology (lead); visualization (equal); writing – original draft (equal). **Marvin A. Ssemadaali:** Investigation (supporting); writing – review and editing (equal). **Harikrishnan Radhakrishnan:** Investigation (supporting); writing – review and editing (equal). **Harold S. Javitz:** Formal analysis (supporting); funding acquisition (supporting); writing – review and editing (equal). **Parijat Bhatnagar:** Conceptualization (lead); funding acquisition (lead); project administration (lead); supervision (lead); visualization (equal); writing – original draft (equal).

## FUNDING INFORMATION

Research reported in this publication was supported in part by the Defense Advanced Research Projects Agency (DARPA) (D19AP00024: DARPA Young Faculty Award, https://www.darpa.mil/work-with-us/for-universities/young-faculty-award), the National Institute of Biomedical Imaging and Bioengineering (DP2EB024245: NIH Director's New Innovator Award Program, https://commonfund.nih.gov/newinnovator), and the National Cancer Institute (R21CA236640, R33CA247739) of the National Institutes of Health (NIH).

## CONFLICT OF INTEREST

All authors disclose no conflict of interest.

## Supporting information


**Appendix S1:** Supporting Information

**Appendix S2:** Supporting Information

## Data Availability

All data used to draw conclusions from our work are present in the article and supporting information.
