# Supplementary material for: Electrically regulated cell‐based intervention for viral infections
Source: Bioeng Transl Med. 2022 Nov 15;8(2):e10434. doi: 10.1002/btm2.10434 (PMC10013824; doi:10.1002/btm2.10434)
Supplement: Supplementary file 1 — Appendix S1: Supporting Information [file BTM2-8-e10434-s002.pdf]

SUPPORTING INFORMATION

Electrically Regulated Cell-based Intervention for Viral Infections

Sherri Newmyer[1], Marvin A. Ssemadaali[1], Harikrishnan Radhakrishnan[1],
Harold S. Javitz[2], Parijat Bhatnagar[1]*

[1]Biosciences Division, [2]Education Division, SRI International
333 Ravenswood Avenue, Menlo Park, CA 94025 USA

Email: Parijat.Bhatnagar@sri.com

Running title: Electrically Responsive Cell Biofactory

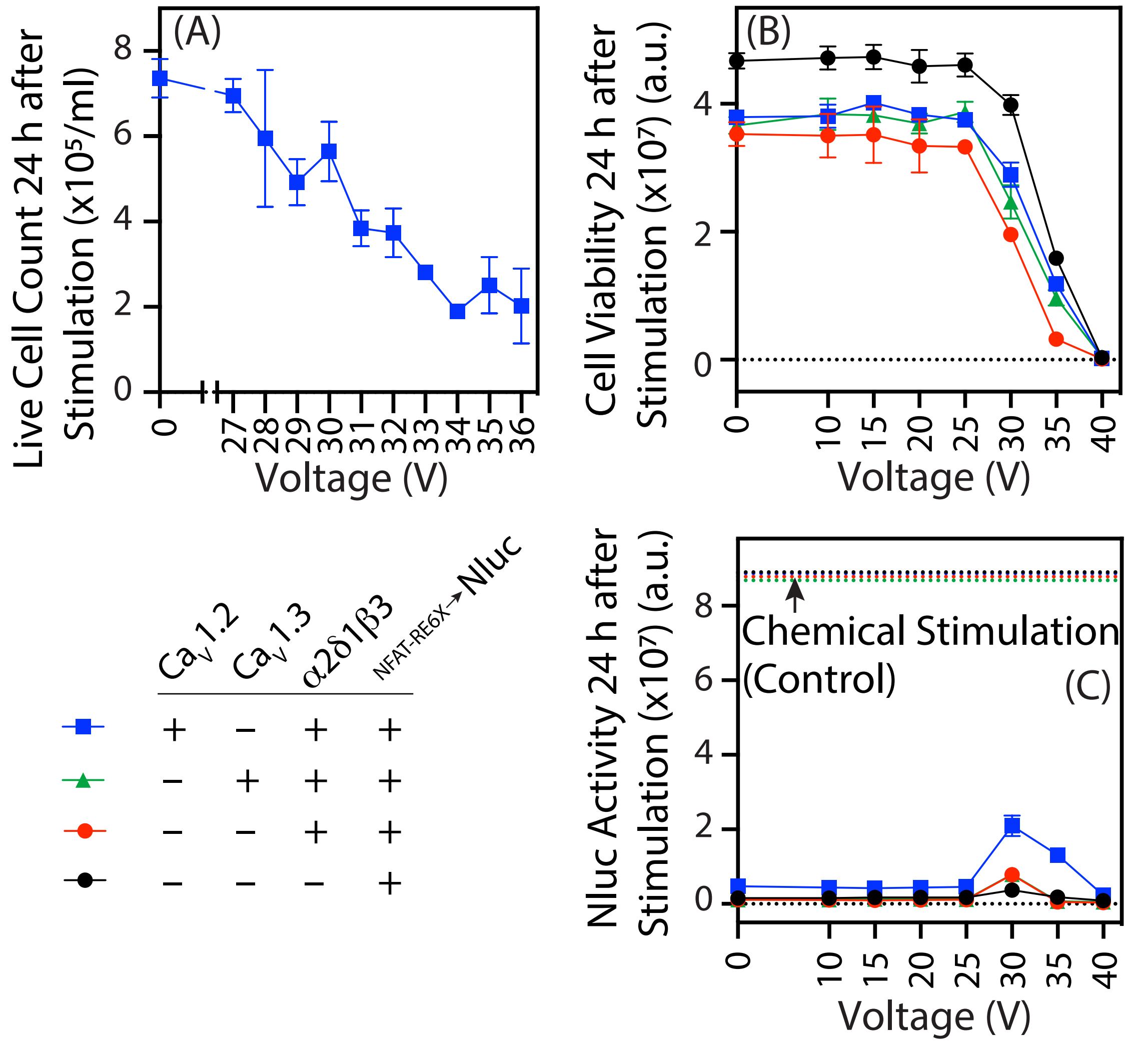

Figure S1. ***Effect of applied voltage on the viability and activation of ES-Biofactory.*** Voltage-gated Ca^{2+} channels ($Ca_V1.2$, $Ca_V1.3$) and auxiliary subunits ($\alpha_2\delta_1$, β_3) were co-engineered into the ES-Biofactory→Nluc platform (Chassis: Jurkat cell). (A-B) Cell Viability and (C) Effector (Nluc) Activity were determined following electrical stimulation of 16,500 initially plated ES-Biofactory→Nluc cells. The applied voltage is indicated on the x-axis. Other parameters included frequency = 20 Hz, pulse duration = 2 msec, stimulus duration = 1 h, and post-stimulus incubation = 24 h. Cell viability, assessed by Trypan Blue Dye Exclusion in (A) and CellTiter-Glo in (B), was determined using n = 2 and n = 4, respectively, with error bars indicate ±1 SD. (C) Experiments indicated a threshold value of 30 V at 20 Hz was necessary to electrically trigger the ES-Biofactory for Effector (Nluc) expression although some compromise in cell viability was observed. Electrically induced activation of the ES-Biofactory was compared to chemically induced stimulation indicated by the dashed lines (30 nM phorbol, 12-myristate, 13-acetate (PMA), 1 µM ionomycin, 24 h). Nluc activity for all observations in (C) was determined using n = 4, error bars indicate ±1 SD.

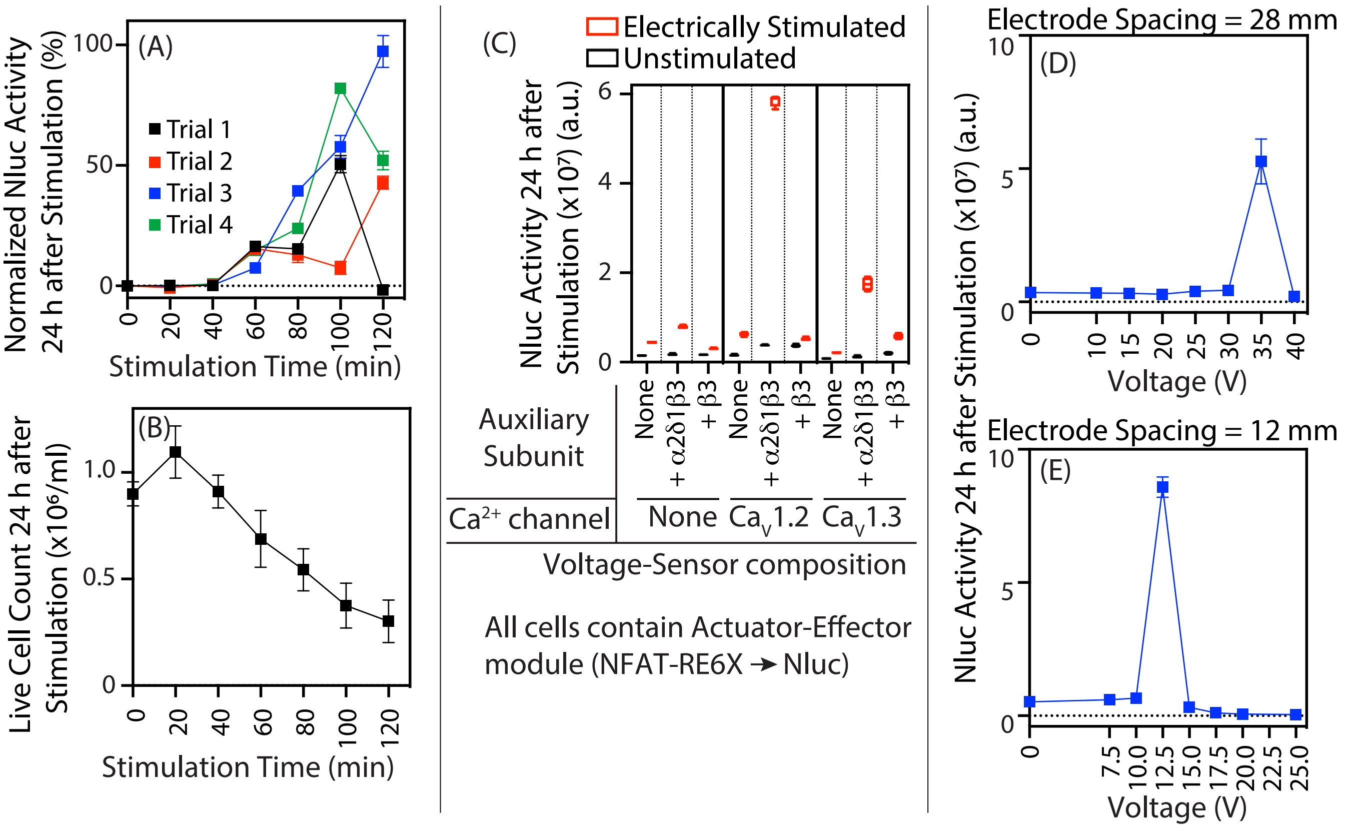

Figure S2. ***Effect of duration of electrical stimulus, expression of voltage-gated Ca^{2+} channels and auxiliary subunits and electrode spacing on the ES-Biofactory.*** Voltage-gated Ca^{2+} channels ($Ca_V1.2$, $Ca_V1.3$) and auxiliary subunits ($\alpha_2\delta_1$, β_3) were co-engineered into the ES-Biofactory→Nluc platform (Chassis: Jurkat cell). Following electrical stimulation, 16,500 of initially plated ES-Biofactory→Nluc cells were assayed for Effector (Nluc) activity and viability ((acridine orange / propidium iodide) (AO/PI)). Unless indicated otherwise on the x-axis, the duration of stimulation was 1 h and the applied voltage = 30 V. All other parameters were kept constant, including frequency = 20 Hz, pulse duration = 2 msec, post-stimulus incubation = 24 h. (A) Effector (Nluc) activity was measured to assess Effector expressed by the ES-Biofactory (Voltage-Sensor: $Ca_V1.2^+\alpha_2\delta_1\beta_3^+$). Nluc activity of the four trials was normalized by setting 0% as the signal for unstimulated cells and 100% as the signal for chemically stimulated cells (30 nM phorbol 12-myristate 13-acetate (PMA), 1 μM ionomycin, 24 h). (B) The AO/PI assay was conducted in parallel to determine cell viability. (A, B) Four trials demonstrated that 60 min of electrical stimulation was required to optimally activate the ES-Biofactory with minimal impact on cell viability. (C) Nluc activity was used to identify the combination of Ca^{2+} channels and auxiliary subunits needed to activate the ES-Biofactory. Results indicate co-expression of $\alpha_2\delta_1\beta_3$ with $Ca_V1.2$ and, to a lesser extent, with $Ca_V1.3$, robustly supported ES-Biofactory activation. (D, E) Two Ion-Optix C-pace lid configurations with (D) 28-mm and (E) 12-mm spaced electrodes were used to stimulate the ES-Biofactory (Voltage-Sensor: $Ca_V1.2^+\alpha_2\delta_1\beta_3^+$). The applied voltage is indicated on the x-axis. Decreasing the inter-electrode distance from 28 to 12 mm proportionately reduced the voltage needed to activate the ES-Biofactory and *Reporter* expression. Nluc activity for all observations in (A, C-E) was determined using n = 4, error bars indicate ±1 SD. Cell viability in (B) values represent the mean of a single AO/PI measurement obtained from the four trials. Error bars indicate ±1 SD.

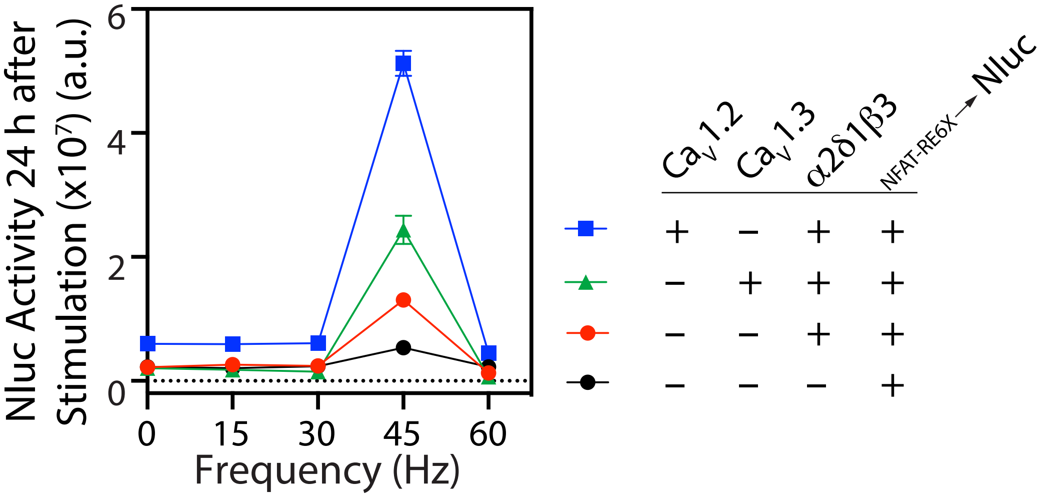

Figure S3. ***Effect of applied voltage and frequency on ES-Biofactory function.*** Following electrical stimulation, 16,500 of initially plated ES-Biofactory→Nluc cells (Voltage-Sensor: $Ca_V1.2^+\alpha_2\delta_1\beta_3^+$, Chassis: Jurkat) were assayed for Effector (Nluc) activity. The applied frequency is indicated on the x-axis. Other parameters included voltage = 20 V, pulse duration = 2 msec, stimulus duration = 1 h, and post-stimulus incubation = 24 h. Data shows optimal stimulation of the ES-Biofactory at 45 Hz frequency when stimulated at 20 V, compared to stimulation with 30 V at 20 Hz (Figure 2C). Nluc activity for all observations was determined using n = 4, error bars indicate ±1 SD.

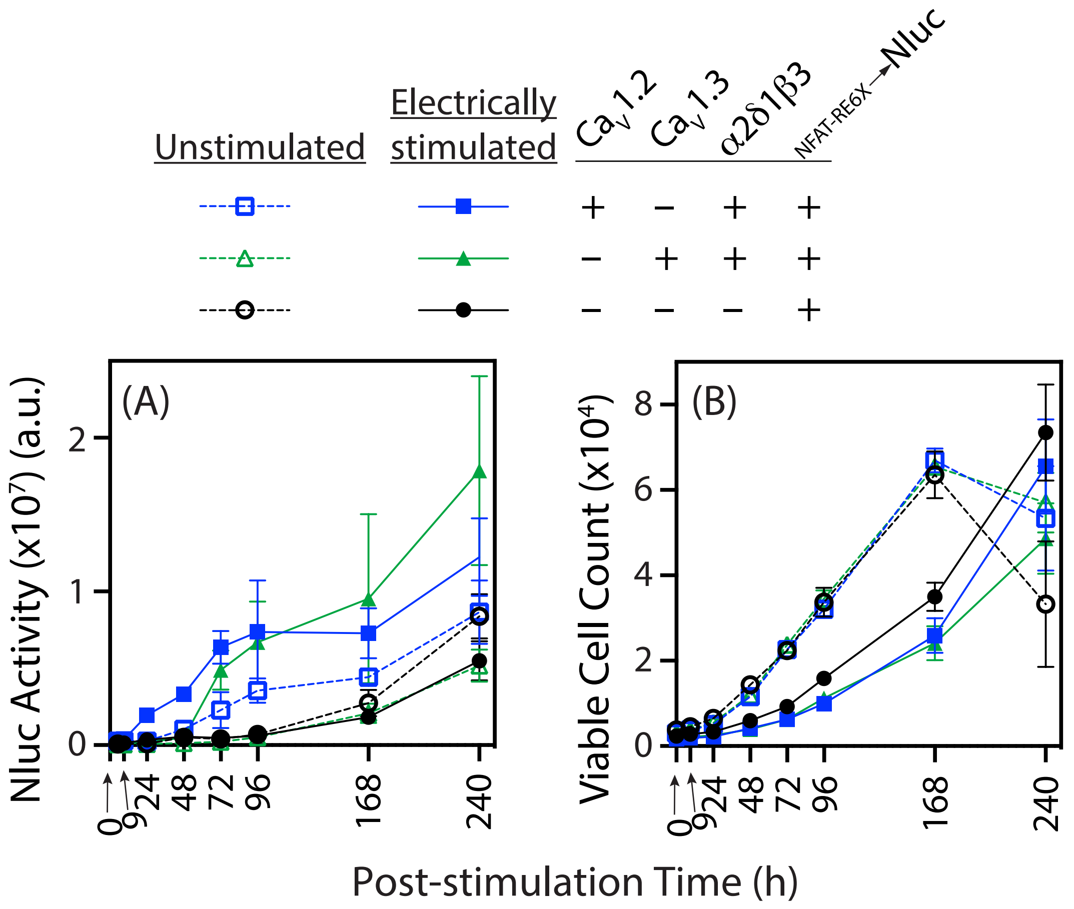

Figure S4. ***Duration of ES-Biofactory expression after electrical stimulation.*** Following electrical stimulation, 650 of initially plated ES-Biofactory→Nluc cells were assayed for Effector activity and viability. The post-stimulus incubation time is indicated on the x-axis. Other parameters included voltage = 30 V, frequency = 20 Hz, pulse duration = 2 msec, and stimulus duration = 1 h. (A) Effector (Nluc) activity and (B) cell count (CellTiter-Glo) were assessed at the indicated time. Effector expression was significantly elevated for both types of ES-Biofactory (Voltage-Sensors – $Ca_V1.2^+\alpha_2\delta_1\beta_3^+$, $Ca_V1.3^+\alpha_2\delta_1\beta_3^+$; Chassis: Jurkat) relative to the unstimulated cells engineered with the same Voltage-Sensor. Leaky Nluc activity increased with cell growth (see time-dependent Nluc activity increase in unstimulated and stimulated Ca_V^{neg} cells, dotted and solid black lines). To isolate Effector expression due to ES-Biofactory activation and independent of cell growth, Nluc activity was normalized to the viable cell count and presented in Figure 2D; normalized Nluc activity = Nluc activity (from panel A) / viable cell count (from panel B). All observations were made using n = 4, error bars indicate ±1 SD.

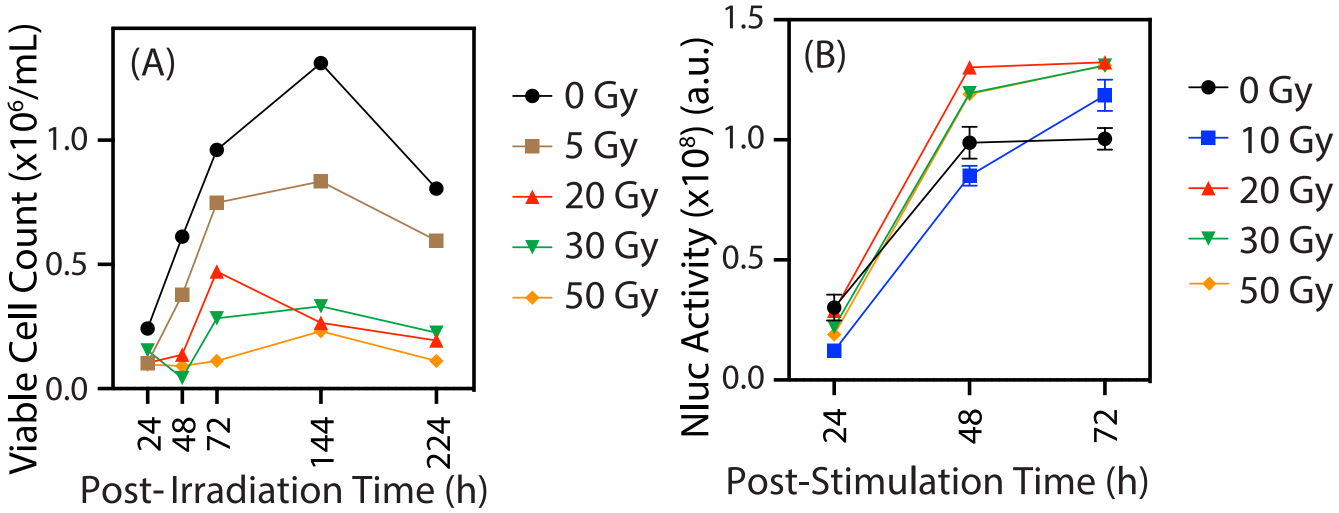

Figure S5. ***Effect of irradiation on cell viability and electrically responsive Effector expression.*** ES-Biofactory→Nluc cells (Chassis: K562) were treated with increasing doses of ionizing radiation as designated in the legend. (A) Following irradiation, the cells were plated at 0.1 x 10^6 cells/ml, and cell viability was measured using AO/PI at the time indicated on the x-axis. The irradiation dose of 30 Gy was effective at limiting cell proliferation over the course of nine days. (B) The irradiated cells were also electrically stimulated to determine the effect of irradiation on electrically driven Effector (Nluc) expression. Following electrical stimulation, 33,000 of initially plated ES-Biofactory→Nluc cells were assayed for Effector (Nluc) activity at 24, 48, and 72 h post-stimulus. Stimulus parameters included voltage = 34 V, frequency = 20 Hz, pulse duration = 2 msec, and stimulus duration = 1 h. Electrical stimulation induced Nluc expression in non-irradiated and irradiated cells, demonstrating that ES-Biofactory→Nluc Effector function is active with irradiation dosing up to 50 Gy. Subsequent experiments used the 30 Gy dose to control cell proliferation while retaining ES-Biofactory Effector function. Nluc activity was calculated using n = 4, error bars indicate ±1 SD.

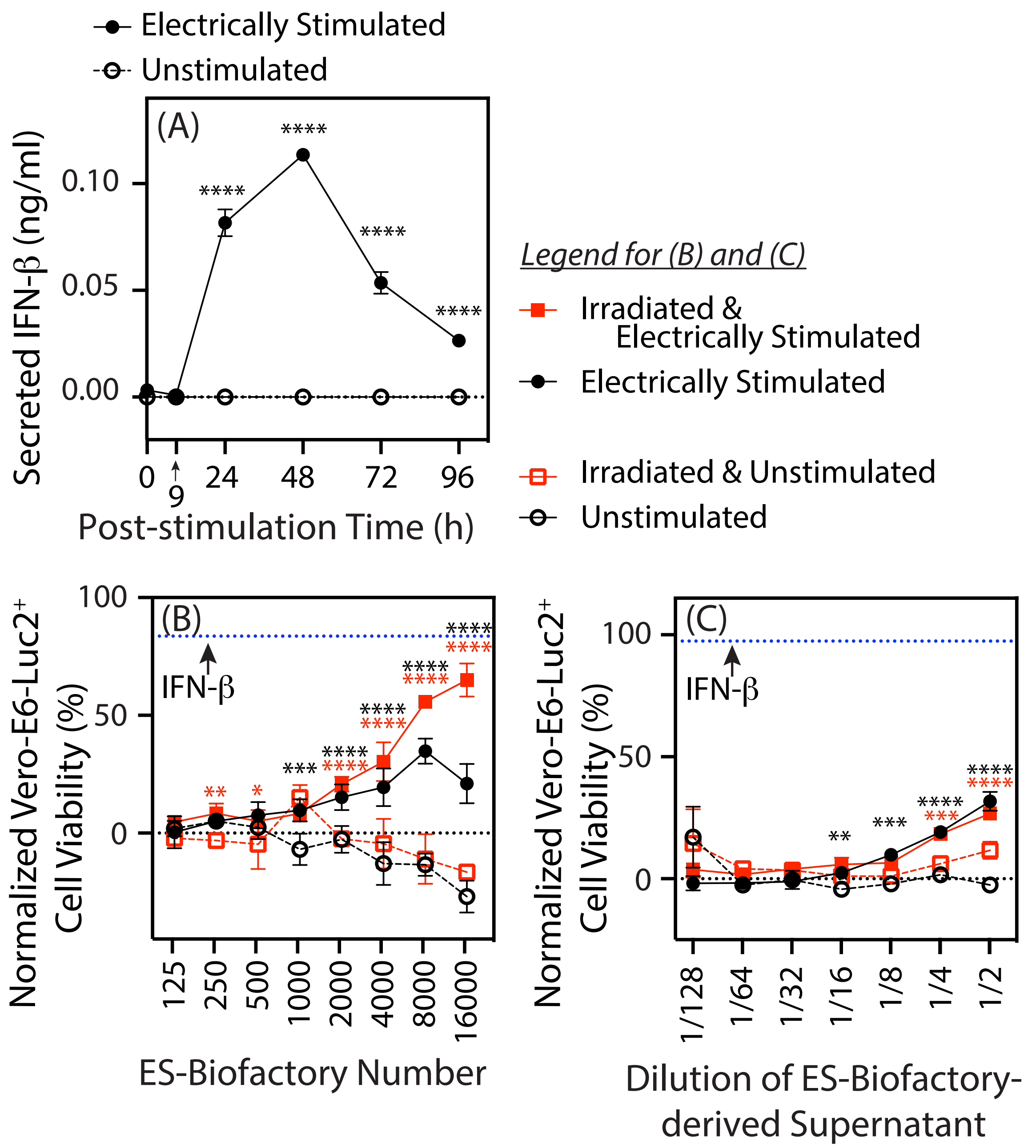

Figure S6. ***Jurkat-based ES-Biofactory driven IFN-β secretion suppresses SARS-CoV-2-mediated host cell killing.*** Following electrical stimulation, ES-Biofactory→IFN-β cells (Chassis: Jurkat) were assessed for (A) Effector (IFN-β) production, (B) therapeutic activity, and (C) prophylactic activity. Electrical stimulation comprised applied voltage = 31 V, frequency = 20 Hz, pulse duration = 2 msec, and stimulation duration = 1 h. (A) A time course of Effector (IFN-β) production was measured by harvesting the supernatant from the ES-Biofactory culture at the designated time point after initiating electrical stimulation and quantifying IFN-β signaling. In response to electrical stimulation, the ES-Biofactory produced IFN-β that increased through 48 h. The antiviral (B) therapeutic and (C) prophylactic activity of the ES-Biofactory-secreted IFN-β was assessed by measuring cell viability in Vero-E6-Luc2^{+} host cells 48 h after SARS-CoV-2 infection and was compared with 0.1 µg (1 µg/mL) of purified IFN-β as a positive control. Cell viability was normalized with 100% representing the luminescence value measured for the untreated, healthy Vero-E6-Luc2^{+} cells and 0% representing that

for the SARS-CoV-2-infected cells. In a therapy treatment protocol (B), the ES-Biofactory→IFN-β cells were electrically stimulated and co-cultured with Vero-E6-Luc2$^+$ cells infected with SARS-CoV-2 prior to the co-culture. In a prophylactic treatment protocol (C), IFN-β containing supernatant was harvested from ES-Biofactory→IFN-β cells 48 h after electrical stimulation. Vero-E6-Luc2$^+$ cells were pretreated with serially diluted IFN-β supernatant prior to SARS-CoV-2 infection. In both protocols, a statistically significant antiviral effect was observed with the electrically stimulated ES-Biofactory→IFN-β treatment compared to the non-stimulated negative controls. The irradiated ES-Biofactory→IFN-β also retained the ability to suppress virus-mediated cell killing. IFN-β production and cell viability for all observations were determined using $n = 3$, error bars indicate ±1 SD. In all panels, the indicated statistical significance of the difference of means was determined with the unpaired two-sample t test with common variance; only differences that were statistically significant at the BKY FDR rate of 0.01 are reported. We note that in contrast to the K562-based delivery of IFN-β (Figure 4C), the Jurkat cell chassis was less effective at prophylactically protecting the Vero-E6-Luc2$^+$ cell (Figure S6C), likely because this cell produced less IFN-β. However, both K562- and Jurkat-based therapeutic treatment preserved cell viability, suggesting that decreased IFN-β dosing is therapeutically effective with sustained application afforded by cell-based delivery.

APPENDIX A

Plasmid A

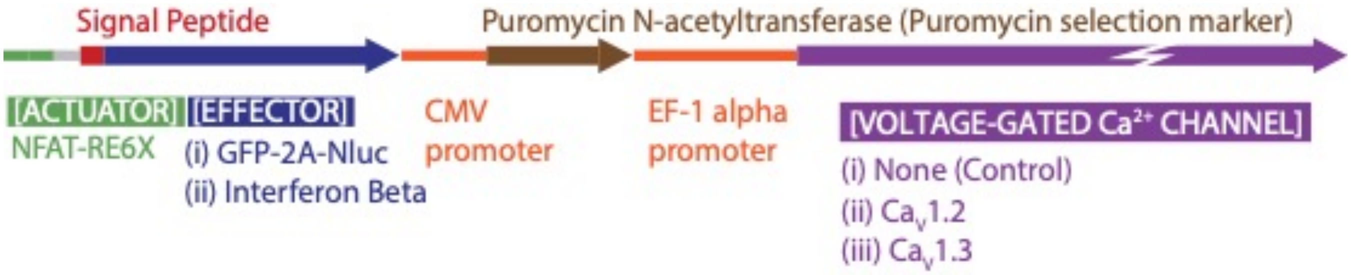

Plasmid A encoded the $Ca_V\alpha_1$ channel of the Voltage-Sensor, the Actuator NFAT-RE6X, and the Effector protein. The $Ca_V\alpha_1$ channel was constitutively expressed. NFAT-RE6X was based on six copies of the NFAT-RE and was placed upstream of the Effector transgene [Ref.(42)]. Effector transgenes, synthesized in response to Ca^{2+} signaling, encoded either the GFP and Nluc reporters or the IFN-β therapeutic protein. Puromycin was used to select for stably transfected cells with Plasmid A.

Plasmid B

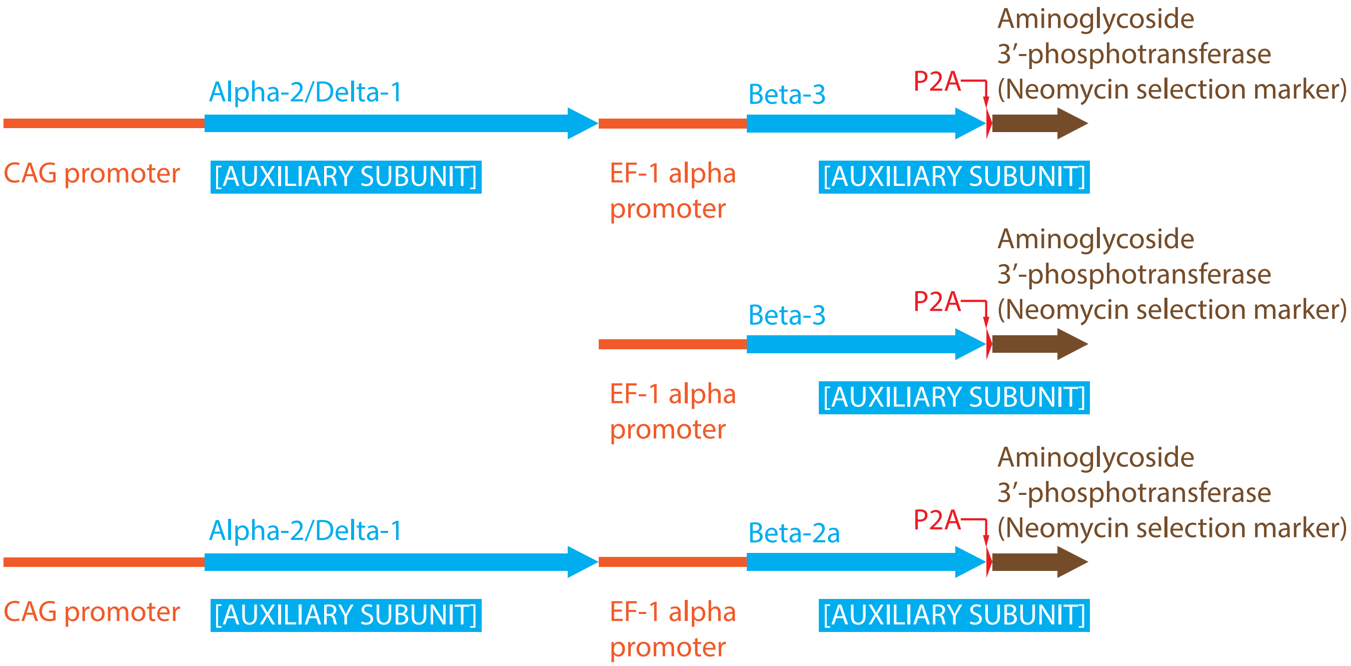

Plasmid B encoded a combination of auxiliary subunits, β_3 alone, or a combination of $\alpha_2\delta_1$ with either β_{2a} or β_3 to support the Voltage-Sensor function of the $Ca_V\alpha_1$ subunit. The auxiliary subunits were constitutively expressed, and neomycin was used to select for cells stably transfected with Plasmid B.
